# Demographic and disease characteristics associated with pain intensity, kinesiophobia, balance, and fall self-efficacy among people with osteoarthritis: a cross-sectional study

**DOI:** 10.1186/s12891-022-05486-4

**Published:** 2022-06-06

**Authors:** Ezinne Chika Ekediegwu, Chigbogu Earnest Akpaenyi, Ifeoma Blessing Nwosu, Ogochukwu Kelechi Onyeso

**Affiliations:** 1 Department of Medical Rehabilitation, Faculty of Health Sciences and Technology, College of Health Sciences, Nnamdi Azikiwe University, Nnewi Campus, Anambra, Nigeria; 2 Prentice Institute for Global Population and Economy, University of Lethbridge, Lethbridge, Alberta Canada; 3 Population Studies in Health, Faculty of Health Sciences, University of Lethbridge, Lethbridge, Alberta Canada

**Keywords:** Demography, Osteoarthritis, Pain measurement, Phobic disorders, Self-efficacy

## Abstract

**Background:**

Osteoarthritis (OA) is a common degenerative joint disease leading to significant pain, mobility limitation, economic burden, reduced quality of life, and disability among adults globally. Psychological factors related to pain intensity (PI), kinesiophobia, fall self-efficacy (FSE), and balance may lead to a poor OA prognosis. This study was designed to explore the association between PI, kinesiophobia, FSE, balance, and age, gender, marital status, site of OA, duration, symmetry, comorbidity, and adaptive behaviours among patients with knee or hip OA.

**Methods:**

This cross-sectional study involved 70 purposively selected participants aged 59.91 ± 11.12 years. Numeric pain rating scale, Tampa scale for kinesiophobia, fall-efficacy scale, and timed up and go test were used to measure PI, kinesiophobia, FSE, and balance, respectively. Statistical analyses were completed with the Pearson correlation test, independent samples t-test, and multiple linear regression.

**Results:**

The participants were mainly women (*n* = 59, 84.3%). However, there was no gender difference in the reported PI, kinesiophobia, FSE, and balance. There was a significant correlation between FSE and balance (*r* = 0.422, *p*<0.001). Kinesiophobia was significantly associated with the presence of comorbidity (β = 0.240, *p* = 0.001) and knee OA (β = 0.208, *p*<0.042). There was an association between FSE and the use of a walking aid (β = ˗0.442, *p*<0.042), stop-for-rest during walking (β = ˗0.292, *p* = 0.002), presence of comorbidity (β = 0.209, *p* = 0.014), and bilateral lower limb OA (β = 0.167, *p* = 0.057). Balance was associated with the use of a walking aid (β = ˗0.421, *p*<0.001) and stop-for-rest during walking (β = − 0.294, *p* = 0.006).

**Conclusion:**

Osteoarthritis-related psychological distress affects both men and women. This study support integration of psychological outcomes in the assessment, management, and follow-up of people with lower limb osteoarthritis. Moreover, comorbidity worsened psychological distress among people with osteoarthritis. Therefore, the traditional biomedical management of osteoarthritis can be optimised by timely diagnosis and treatment of comorbidities, and the inclusion of psychotherapy.

## Introduction

Osteoarthritis (OA) is a chronic degenerative disease of the articular cartilages of synovial joints [1, 2]. The knee is the most frequently affected joint, followed by the hand and hip [3]. The combined impact of knee and hip OA was ranked as the eleventh leading cause of disability out of 291 diseases, globally [4]. The most common symptoms of OA are joint pain and stiffness [4, 5]. The symptoms may be asymmetric at onset, but the majority of the sufferers will eventually develop bilateral OA [5, 6]. Each additional joint affected by OA results in a decrease in physical function, and an increase in psychological distress [7] and overall disease burden [6].

As of 2019, the global cases of the knee and hip OA were approximately 364.6 million and 33 million, respectively, with an estimated annual increase rate of 0.3% for both types [3]. A recent systematic review reported the pooled global prevalence of knee OA in individuals aged 40 years and above as 22·9% [8], while 19.6 to 20.6% were reported by independent studies conducted among Nigerians of the same age range [9]. Nonetheless, a systematic review of OA prevalence in Africa revealed the paucity of data on the prevalence of hip OA in Nigeria [10]. Although the overall prevalence of OA in Nigeria was lower than the global average [3, 11], the estimated increase in cases from 1999 to 2019 was between 100 and 150% [3]. Therefore, there is a need to examine the OA demographic and disease characteristics in Nigeria and strategise for prevention and care. The care for people with OA in Nigeria starts with general practitioners who make a provisional diagnosis of OA, prescribe medications, and refer the patients to specialists [12]. Specialist care is usually accessible at various tertiary hospitals through the services of consultant orthopaedic doctors who reassess the patients, review their medications, refer them for allied health care such as physiotherapy, and schedule surgery when necessary [12, 13]. Therefore, OA management in Nigeria is mostly biomedical, as most patients do not have access to psychosocial therapy.

Physical, biological, cognitive, behavioural, social, and occupational factors have been associated with a poor prognosis of lower limb OA [14, 15]. However, the impacts of several maladaptive psychological factors such as fear of movement, fear of falling, and perceived imbalance are less studied [7, 16]. The majority of people with lower limb OA develop kinesiophobia – an excessive, irrational, and debilitating fear of physical movement and activity due to a feeling of vulnerability to pain, injury, or re-injury [17, 18]. Kinesiophobia hinders movement and exercise therapy and promotes stiffness, pain, sedentary behaviour, and disability [17, 19]. Fall self-efficacy (FSE) has been defined as the perceived self-confidence in avoiding falls during essential non-hazardous activities [20]. It is a measure of fear of falling, the psychological sequel of falls and perceived fall risk [21]. Balance is considered a key component in many activities of daily living, from simple activities such as quiet standing to more complex activities such as change of direction while walking [22]. The pain and joint structural changes resulting from OA may compromise the musculoskeletal system leading to perceived or real imbalance, fear of falling, and kinesiophobia [9, 23].

Osteoarthritis Research Society International (OARSI) opined that people with OA experience more pain, fatigue, levels of disability and activity limitation than people of comparable age [24]. Therefore, lower limb OA coupled with kinesiophobia, imbalance, and low FSE can result in higher activity limitation, disability, and a sedentary lifestyle that predisposes an individual to other non-communicable diseases [18, 25–27]. Lentz and colleagues suggested that more psychological studies and interventions are needed to augment the biomedical interventions which do not often address psychological distress that influences OA-related pain and disability [7]. Therefore, many developed countries have started incorporating psychosocial well-being in OA management [28, 29]. However, there is a paucity of studies on the demographic and disease characteristics that could predict what sorts of patients are at risk for worse kinesiophobia, FSE, and perceived balance in Nigeria. The present study is necessary to inform stakeholders of the impact of psychological factors among people with OA in a low-resource setting such as Nigeria.

Therefore, this study examined the levels of pain, FSE, kinesiophobia, and balance among lower limb OA patients attending tertiary hospitals in Anambra, Nigeria. The authors hypothesised that there will be no significant (a) gender difference in pain intensity (PI), FSE, kinesiophobia, and balance, (b) correlation among PI, FSE, kinesiophobia, and balance, and (c) association between demographic and disease factors with each of FSE, kinesiophobia, and balance among the participants.

## Methods

### Study design

The study was a cross-sectional study of 70 ambulatory patients with knee OA, hip OA, or both who were receiving treatment in tertiary hospitals in Anambra, Nigeria. The study protocol was approved by the Human Research Ethics Committees of the Faculty of Health Sciences and Technology, Nnamdi Azikiwe University, Anambra, Nigeria (NAU/FHST/2021/MRH103). All the eligible participants signed an individual informed consent form before partaking in the study. Participants were informed of their right to withdraw at any point in the study. The approved protocol, participants’ privacy, and confidentiality of data were strictly adhered to.

### Setting

The study was conducted in the outpatient orthopaedic clinics of the tertiary hospitals in Anambra, Nigeria, between February and July 2021. The tertiary hospitals were referral centres that offered comprehensive orthopaedic care including prescription of medications, braces, and walking aids, intra-articular injection, surgical consultation, and referral to allied services such as physiotherapy. All the study participants were diagnosed by consultant orthopaedic doctors in each hospital. The diagnostic criteria included positive medical history and physical examination, and the presence of osteophytic changes in a plain radiograph based on Kellgren-Lawrence’s grades two to four [24, 30]. Diagnosed outpatients who were receiving care at the clinics were introduced to the researchers by the nurses during a follow-up visit. A consecutive sampling technique was used to recruit participants who met the study eligibility criteria.

### Participants and eligibility criteria

Participant inclusion criteria were being (a) diagnosed with knee or hip OA, (b) attending follow-up orthopaedic appointments in any of the tertiary hospitals, (c) fluent in the English language, and (d) willing to grant an informed consent and participate in the study procedures.

Participants were excluded if they were below 18 years of age, non-ambulant, diagnosed with other forms of arthritis beyond OA, other neurological and musculoskeletal disorders affecting the lower limbs, or OA patients with acute hip or knee pain following trauma. Patients with systemic diseases such as sickle cell disease, complex regional pain syndrome, cancer, chronic kidney disease, cardiovascular diseases, uncontrolled diabetes, or patients who had arthroplasty or intra-articular corticosteroid injection within 1 month before the study were also excluded.

### Variables

With the permission of the individual hospitals, the authors extracted relevant sociodemographic and disease history from the medical records of patients who met eligibility criteria and granted informed consent to participate in the study. Therefore, participants’ age, gender, marital status, pain duration, walking aid, comorbidity, walking behaviour, and site of OA were recorded and crosschecked on physical examination. The primary outcomes: PI, kinesiophobia, FSE, and balance were assessed with appropriate instruments and recoded as continuous variables. The secondary outcomes: age (years) and pain duration (weeks) were recorded as continuous variables. Marital status (single = 1, married = 2, divorced/separated = 3, widowed = 4) was treated as nominal variable. Walking aid (frames = 1, crutches = 2, canes = 3) was considered as an ordinal variable based on decreasing levels of support. Gender (female = 0, male = 1), comorbidity, stop-to-rest during walking, and site of OA were recorded as dichotomous variables (absent = 0, present = 1).

### Research instruments and procedures for data collection

The Numeric Pain Rating Scale (NPRS) was used to obtain participants’ PI on a scale of 1 (no pain) to 10 (worst imaginable pain). The NPRS is a convenient, valid, reliable, and responsive measure of pain intensity devoid of interference from non-pain factors among people with OA [31]. The interclass correlation reliability, *r* = 0.95, criterion validity using visual analogue scale = 0.94 [31].

Afterwards, the participants were administered two questionnaires: the Tampa scale for kinesiophobia (TSK) and the fall-efficacy scale. The TSK is a self-completed 17-item questionnaire used to assess the subjective rating of fear of movement [18]. Each item is rated on a 4-point Likert scale (strongly disagree = 1 to strongly agree = 4), the total score ranges from 17 to 68, and higher scores (37 and above) indicate an increasing degree of kinesiophobia; the cut-off score was developed by Vlaeyen and colleagues [32]. The test-retest reliability of TSK ranged from *r* = 0.64 to 0.80 [33].

The fall-efficacy scale is a 10-item questionnaire; each item is rated on a scale of 1 (confidence) to 10 (no confidence in these activities). Out of a total score of 100, scores of 70 and above indicate the individual has a fear of falling [20]. The fall-efficacy scale is a valid and reliable instrument (test-retest reliability, *r* = 0.91) [20].

Finally, each participant’s balance was assessed through the timed up and go (TUG) test – a simple, sensitive, and specific screening test for risk of falls, dynamic balance, lower extremity function, and mobility [34]. Following the recommendation of an advisory group comprised of 138 experienced clinicians and researchers from 16 countries, OARSI endorsed five performance-based tests including TUG, for physical function assessment among people with knee or hip OA [35]. The TUG was recommended for this type of study ahead of the Berg balance test and the Dynamic Gait Index due to its ability to capture the domains necessary for dynamic stability [34]. The TUG was completed by monitoring the participant, rising from an armchair, walking a distance of three metres, and returning to the chair unaided. The time taken to complete the task was recorded using a stopwatch (Kadio, made in China). The intra- and inter-tester reliability of TUG ranged from = 0.92 to 0.99 [36]. People with lower limb OA who takes ≥13.5 seconds to complete the TUG are at risk of falling [37].

All the data collected through the medical records, physical examination, NPRS, TUG, TSK, and fall-efficacy scale were anonymised, entered into a password-protected electronic spreadsheet, and stored in a flash drive accessible to only the authors.

### Bias

To avoid sampling bias, participant recruitment and data collection from the tertiary hospitals were conducted simultaneously by the authors via consecutive sampling. Consecutive sampling is a type of nonprobability sampling technique in which every subject meeting the inclusion criteria is selected until the required sample size is achieved. It is better than convenience sampling in controlling sampling bias [38]. None of the patients who meet the inclusion criteria declined participation.

### Sample size determination

The sample size for the present study was calculated using the G*Power 3.1.9.4 software. A sample of 70 participants was appropriate for a 9-predictor multiple linear regression given a moderate effect size of 0.3, error of probability = 0.05, and power = 95.0%.

### Data analysis

Data were analysed using the statistical package for social sciences (SPSS) version 26. Descriptive statistics such as frequency, percentage, mean, and standard deviation were used to summarise the participants’ demographic and disease characteristics. Inferential statistics – independent samples t-test was used to analyse the gender differences in PI, kinesiophobia, FSE, and balance. The correlations among the outcome variables were completed using Pearson’s correlation coefficient. A forward stepwise, multiple linear regression was used to determine the demographic and disease factors that best predict kinesiophobia, FSE, and balance. The predictors were age, gender, marital status, PI, pain duration, the site of OA, walking aid, presence of comorbidity, cognitive function, and stop-for-rest during walking. The data were diagnosed and fixed for missing values, univariate and multivariate outliers, normality, linearity, and multicollinearity. Alpha level was set at 0.05.

## Results

### Participant’s demographic and disease characteristics

Seventy participants aged 59.91 ± 11.124 completed the study. Table 1 shows that many of the participants were women (*n* = 59, 84.3%) of 61 years and above (*n* = 34, 48.6%) who had at least one comorbidity (*n* = 31, 44.3%). All the 70 (100%) participants had painful symptomatic OA. Many of the participants 61 (87.1%) had suffered OA for more than 6 months. Specifically, 43 (61.4%) participants suffered knee OA, 18 (25.7%) suffered hip OA, and 9 (12.9%) suffered both knee and hip OA. In terms of the side of the body involved, 18 (25.7%) and 8 (11.4%) participants reported left and right lower limbs, respectively. About three-quarters of the participants (55, 78.6%) did not need a walking aid (Table 1).

**Table 1:** Participants’ demographic and disease characteristics

Parameter	***Frequency*** (%)
**N**	70 (100)
**Gender**	
Male	11 (15.7)
Female	59 (84.3)
**Age Range in years**	
18–30	2 (2.9)
31–40	5 (7.1)
41–50	12 (17.1)
51–60	17 (24.3)
60 and above	34 (48.6)
**Marital Status**	
Single	4 (5.7)
Married	47 (67.1)
Divorced	1 (1.4)
Widowed	18 (25.8)
**Pain due to OA**	
Yes	70 (100)
No	0 (0)
**Type of OA**	
Knee OA	43 (61.4)
Hip OA	18 (25.7)
Both Knee and Hip OA	9 (12.9)
**Site of OA**	
Left Lower Limb	18 (25.7)
Right Lower Limb	8 (11.4)
Both Lower Limbs	44 (62.9)
**Duration**	
Less than 3 weeks	3 (4.3)
3 weeks to 6 months	6 (8.6)
More than 6 months	61 (87.1)
**Comorbidity**	
Yes	31 (44.3)
No	39 (55.7)

*OA* Osteoarthritis

### Univariate analyses

The average PI was 6.0 ± 1.6 (NPRS, 0 to 10). Similarly, the average time of completion of the TUG test (22.3 ± 37.8 seconds) was above the benchmark (≤13.5 seconds) [36]. Also, the average kinesiophobia score (42.4 ± 6.7) was higher than the pathological benchmark (> 37) [32]. However, FES (22.6 ± 19.4) was within the normal range (< 70) [20].

### Bivariate analyses

Table 2 shows no significant gender difference in PI, kinesiophobia, FSE and balance. There was a significant positive correlation between FSE and TUG scores (*r* = 0.422, *p* < 0.001). There was no significant correlation between other pairs of psychological outcomes (Table 3).

**Table 2:** Gender differences in the psychological parameters and balance

Parameters	Gender *Mean* ± *SD*			Mean Difference (MD)	t-statistic	***p***-value
	Total	Male	Female			
**Pain Intensity**	6.0 ± 1.6	6.09 ± 1.30	5.98 ± 1.67	0.11	0.241	0.813
**Kinesiophobia**	42.4 ± 6.7	43.73 ± 6.97	42.14 ± 6.72	1.59	0.699	0.496
**Fall Self-Efficacy**	22.6 ± 19.4	24.45 ± 18.38	22.29 ± 19.74	2.16	0.355	0.728
**TUG Score**	22.3 ± 37.8	17.09 ± 7.99	23.27 ± 35.50	−6.18	1.185	0.240

*TUG* Timed up and go (balance) score

**Table 3:** A matrix table showing Pearson’s correlation among pain intensity, kinesiophobia, fall self-efficacy, and timed up and go scores

Parameters	Kinesiophobia Score *r* (*p*-value)	Fall Self-Efficacy Score *r* (*p*-value)	TUG Score *r (p*-value)
**Pain Intensity**	0.086 (0.480)	0.137 (0.259)	0.099 (0.414)
**Kinesiophobia**	_	0.139 (0.252)	- 0.145 (0.231)
**Fall Self-Efficacy**	_	_	0.422 (< 0.001) *

*OA* Osteoarthritis, *TUG* Timed up and go (balance) score

* *r* was significant at *p* < 0.05

### Multivariate analyses (predictors of kinesiophobia, fall self-efficacy, and balance)

Forward stepwise multiple linear regression (Table 4) showed that the best set of predictors for kinesiophobia were presence of comorbidity (Standardised Regression Coefficients (β) = 0.240, *p* < 0.001) and knee OA (β = 0.208, *p* < 0.042). Predictors for fall self-efficacy were use of walking aid (β = − 0.442, p < 0.001), stop-for-rest during walking (β = 0.292, *p* = 0.002), knee OA (β = 0.241, *p* = 0.006), comorbidity (β = 0.209, *p* = 0.014), and bilateral limb OA (β = 0.167, *p* = 0.057). Predictors for balance were use of walking aid (β = − 0.421, p < 0.001), and stop-for-rest during walking (β = 0.294, *p* = 0.006). The model summaries were provided as foot note of Table 4.

**Table 4:** Multiple linear regression: predictors of kinesiophobia, fall self-efficacy and perceived balance

Subject	Best set of Predictors	Regression Coefficients (B)	Standardised Regression Coefficients (β)	***p***-value
**Kinesiophobia**	(Constant)	38.834	_	< 0.001
	Comorbidity	3.233	0.240	< 0.001*
	Knee OA	2.850	0.208	0.042*
**Fall self-efficacy**	(Constant)	42.291	_	< 0.001
	Walking aid	−7.959	−0.422	< 0.001*
	Stop-for- rest	16.801	0.292	0.002*
	Knee OA	9.531	0.241	0.006*
	Comorbidity	8.102	0.209	0.014*
	OA on both legs	6.668	0.167	0.057
**Timed up and go**	(Constant)	79.880	_	< 0.001
	Walking aid	−13.398	−0.421	< 0.001*
	Stop-for-rest	28.532	0.294	0.006*

Model Summaries:

Kinesiophobia: *R* = 0.32; F (2, 67) = 3.770, *p* = 0.028

Fall self-efficacy: *R* = 0.75; F (5, 64) = 16.587, *p* < 0.001

Timed up and go: *R* = 0.58; F (2, 67) = 1.704, *p* < 0.001

*OA* Osteoarthritis

* β significant at *p* < 0.05

## Discussion

This study explored the extent and correlates of balance, fall self-efficacy, and fear of movement among people with osteoarthritis. Although strenuous activities can flare up lower limb OA [39], avoidance of movement can lead to a vicious cycle of pain and joint stiffness [25, 40]. Therefore, experts prescribe moderate physical activities such as exercises in the management of OA [9, 41]. However, psychological factors relating to fear, perceived balance, and fall efficacy may deter people with OA from engaging in prescribed exercises and other activities of daily living [17, 27, 40]. We found no significant association between demographic variables such as age, gender, marital status, or pain duration with kinesiophobia, FSE, and balance. However, disease characteristics such as the joint involved (knee OA), bilateral presentation, comorbidity, and adaptive behaviours such as the use of walking aid and stop-for-rest during walking had a significant association with kinesiophobia, FSE, and balance.

All the study participants reported joint pain in at least one of their lower limbs. The univariate analyses showed that participants had moderate pain, balance limitation, and fear of movement, but low fear of falling. The bivariate analysis showed a significant positive correlation between fear of falling and imbalance. This implies that people with higher fear of falling spent more time completing the TUG test. The outcome concurred with a previous study that reported a positive correlation between fear of falling and postural instability [42]. However, there was no significant correlation between pain intensity and other primary outcomes: balance, fear of movement, and fear of falling. This result suggests that balance, fears of falling and movement may not be solely associated with patients’ pain intensity but could be psychological. The outcome of this study supported other scholars who opined that psychological interventions were needed to augment the biomedical management of OA [7, 18, 28, 39]. Osteoarthritis care in Nigeria is mainly biomedical, Osage and Yakubu’s clinical audit of OA care in a tertiary hospital in Nigeria showed that none of the targeted treatment standards was met, moreover, psychological interventions were not administered [43].

The regression models showed comorbidity, use of walking aid, stop-for-rest while walking, and site of OA as important predictors of balance and psychological functionality among people with OA. Specifically, comorbidity and knee OA were significant predictors of fear of movement. A recent UK-based combined case-control and cohort study reported a strong association between OA and comorbidities [26]. Another study found a strong correlation between knee OA and fear of movement [18]. The predictors of fear of falling included knee OA, comorbidity, and bilateral OA. Others were the use of walking aid and stop-for-rest while walking which were also predictors of balance. This further confirmed the bivariate correlation between fear of falling and balance. Walking aid users and people who required rest breaks while walking had fear of falling, however people who required rest breaks achieved more dynamic balance than walking aid users. This outcome aligned with the study of Champagne et al. [42], where a correlation was observed between fall-related efficacy and postural steadiness (balance). Moreover, the regression model showed that bilateral OA is a predictor of fear of falling. Each additional joint affected by OA results in a decrease in physical function and an increase in psychological instability [7] and overall disease burden [6] such as a high propensity for a sedentary life, comorbidities, dependency, use of walking aids, and accidental fall [26, 27, 44]. Task modifications such as stop-for-rest during walking may be perceived as a mobility disability, but it helps community-dwelling older people to improve their outdoor life [45]. Further research is needed to determine whether we should advise patients with bilateral OA to adopt intermittent rest while walking.

The present study agreed with the global data that OA of the knee is more prevalent than hip OA [3]. We also observed that about 12.9% of the participants had both knee and hip OA. This finding has shown that concurrent knee and hip OA is common in the study population. Our research was not a prevalence study, but we found a huge gap in the literature concerning the prevalence of lower limb OA among community-dwelling Nigerians. The most cited community-based knee OA prevalence in Nigeria was published 14 years ago [46]. A recent systematic review of OA prevalence in Africa found no community-based study on the prevalence of hip OA in Nigeria, the only available literature was a hospital-based study published 27 years ago [10]. However, our participants’ characteristics were similar to other Nigerian studies on hospital-attending OA patients [12, 27]. Demographic findings in the present study showed that the majority of the participants were women and older persons. This observation agreed with Hootman et al. [47], who asserted that the prevalence of doctor-diagnosed OA was higher in older age groups, rising from 24.3% in patients aged 45 years old to 47.4% in patients aged 65 years. This result also concurred with other studies [12, 27, 48] that reported female gender and older age as risk factors for OA. Some experts attributed this phenomenon to the well-established imbalance in bone homeostasis among post-menopausal women and the general biophysical decline in older people [49].

The clinical implication of this study is that incorporating fall self-efficacy, TUG, and kinesiophobia tests in the assessment and follow-up of people with OA may provide a holistic biopsychological status of the patient. The traditional biomedical management of lower limb OA could be enhanced by timely diagnosis and treatment of other comorbidities and the inclusion of psychological assessment and psychosocial therapy. Modified physical activities such as stop-for-rest during walking may provide a reasonable alternative to absolute sedentary life and increase the potential for outdoor activities among OA patients [44, 50].

### Limitations

The study participants were sampled by a nonprobability (consecutive) method – affecting the generalisability of our findings. However, the demographic findings in the present study were similar to two previous studies conducted among people with OA in different regions of the country [12, 27]. Moreover, as with most survey designs, we relied on self-reported information. Our participants were predominantly women. Although the student t-test can handle sample size differences between two independent groups, an age- and gender-matched randomised sample would be necessary to make valid gendered inferences on the psychological factors associated with OA. Another limitation of this study was that we could not collect data on the types, numbers, and severity of the comorbidities. A multi-centre study with a national representative sample may be needed for a more generalisable result.

## Conclusion

Demographic factors such as age, gender, marital status, and disease duration were not determinants of kinesiophobia, fall self-efficacy, or balance among people with lower limb osteoarthritis. However, this study has shown some modifiable factors such as comorbidity, use of walking aid, and adaptive walking behaviours that can be manipulated to mitigate psychological limitations and improve mobility among patients with lower limb osteoarthritis. The presence of knee osteoarthritis and comorbidity was associated with high fear of falling and low fall self-efficacy, but no significant association with balance score. Use of walking aid was associated with reduced fear of falling, but a low balance score. Stopping occasionally to rest while walking was associated with higher fall self-efficacy and balance scores.

## Data Availability

The dataset generated and analysed during the current study will be made public by 2026 but is available from the corresponding author on reasonable request. This is in adherence to our institutional policy of holding data for 5 years after study completion.

## Abbreviations

FSE: Fall Self-efficacy
NPRS: Numeric Pain Rating Scale
OA: Osteoarthritis
OARSI: Osteoarthritis Research Society International
PI: Pain Intensity
TSK: Tampa Scale for Kinesiophobia
TUG: Time Up and Go
UK: United Kingdom

## References

[1] Mustonen AM, Nieminen P (2021). Extracellular vesicles and their potential significance in the pathogenesis and treatment of osteoarthritis. Pharmaceuticals (Basel).

[2] Gates LS, Leyland KM, Sheard S, Jackson K, Kelly P, Callahan LF (2017). Physical activity and osteoarthritis: a consensus study to harmonise self-reporting methods of physical activity across international cohorts. Rheumatol Int.

[3] Long H, Liu Q, Yin H, Wang K, Diao N, Zhang Y, et al. Prevalence trends of site-specific osteoarthritis from 1990 to 2019: findings from the Global Burden of Disease Study 2019. Arthritis Rheumatol. 2022. 10.1002/art.42089 PMC9543105 35233975

[4] Cross M, Smith E, Hoy D, Nolte S, Ackerman I, Fransen M (2014). The global burden of hip and knee osteoarthritis: estimates from the global burden of disease 2010 study. Ann Rheum Dis.

[5] National Institute of Arthritis and Musculoskeletal and Skin Diseases. Osteoarthritis. https://www.niams.nih.gov/health-topics/osteoarthritis (2015). Accessed on 14 Mar 2022.

[6] Metcalfe AJ, Andersson ML, Goodfellow R, Thorstensson CA (2012). Is knee osteoarthritis a symmetrical disease? Analysis of a 12 year prospective cohort study. BMC Musculoskelet Disord.

[7] Lentz TA, George SZ, Manickas-Hill O, Malay MR, O'Donnell J, Jayakumar P (2020). What general and pain-associated psychological distress phenotypes exist among patients with hip and knee osteoarthritis?. Clin Orthop Relat Res.

[8] Cui A, Li H, Wang D, Zhong J, Chen Y, Lu H (2020). Global, regional prevalence, incidence and risk factors of knee osteoarthritis in population-based studies. EClinicalMedicine..

[9] Adhama AI, Akindele MO, Ibrahim AA (2021). Effects of variable frequencies of kinesthesia, balance and agility exercise program in adults with knee osteoarthritis: study protocol for a randomized controlled trial. Trials..

[10] Usenbo A, Kramer V, Young T, Musekiwa A (2015). Prevalence of arthritis in Africa: a systematic review and meta-analysis. PLoS One.

[11] Fatoye F, Gebrye T, Fatoye C, Mbada C, Olaniyi H, Oyeleye O (2020). PMS30 real world prevalence, pattern and cost of physiotherapy treatment for osteoarthritis in Nigeria. Value Health.

[12] Adebusoye LA, Ogunbode AM, Alonge TO (2013). Magnitude of knee osteoarthritis and associated risk factors among adult patients presenting in a family practice clinic in Nigeria. J Med Trop.

[13] Abang I, Anisi C, Asuquo J, Agweye P, Ngim N, Mpama E (2019). Osteoarthritis: knowledge and acceptability of total joint replacement. Age..

[14] Cimmino MA, Ferrone C, Cutolo M (2011). Epidemiology of chronic musculoskeletal pain. Best Pract Res Clin Rheumatol.

[15] Green DJ, Lewis M, Mansell G, Artus M, Dziedzic KS, Hay EM (2018). Clinical course and prognostic factors across different musculoskeletal pain sites: a secondary analysis of individual patient data from randomised clinical trials. Eur J Pain.

[16] Gorczyca R, Filip R, Walczak E (2013). Psychological aspects of pain. Ann Agric Environ Med.

[17] Vlaeyen JWS, Linton SJ (2000). Fear-avoidance and its consequences in chronic musculoskeletal pain: a state of the art. Pain..

[18] Aykut Selçuk M, Karakoyun A (2020). Is there a relationship between kinesiophobia and physical activity level in patients with knee osteoarthritis?. Pain Med.

[19] Lundberg M, Styf J, Jansson B (2009). On what patients does the Tampa scale for Kinesiophobia fit?. Physiother Theory Pract.

[20] Tinetti ME, Richman D, Powell L (1990). Falls efficacy as a measure of fear of falling. J Gerontol.

[21] Jørstad EC, Hauer K, Becker C, Lamb SE (2005). ProFaNE group. Measuring the psychological outcomes of falling: a systematic review. J Am Geriatr Soc.

[22] Dunsky A, Zeev A, Netz Y (2017). Balance performance is task specific in older adults. Biomed Res Int.

[23] Gupta G, Sharma A (2018). Impact of osteoarthritis on balance, perceived fear of fall and quality of life. Orthop Rheumatol Open Access J.

[24] Osteoarthritis Research Society International. Osteoarthritis: a serious disease, submitted to the U.S. Food and Drug Administration on December 1, 2016. https://oarsi.org/education/oarsi-resources/oarsi-white-paper-oa-serious-disease. Accessed on 14 Mar 2022.

[25] De Vroey H, Claeys K, Shariatmadar K, Weygers I, Vereecke E, Van Damme G (2020). High levels of kinesiophobia at discharge from the hospital may negatively affect the short-term functional outcome of patients who have undergone knee replacement surgery. J Clin Med.

[26] Swain S, Coupland C, Mallen C, Kuo CF, Sarmanova A, Bierma-Zeinstra SMA (2021). Temporal relationship between osteoarthritis and comorbidities: a combined case control and cohort study in the UK primary care setting. Rheumatology (Oxford).

[27] Odole A, Ekediegwu E, Ekechukwu END, Uchenwoke C (2019). Correlates and predictors of pain intensity and physical function among individuals with chronic knee osteoarthritis in Nigeria. Musculoskelet Sci Pract.

[28] Tan BY, Thach T, Munro YL, Skou ST, Thumboo J, Car J (2021). Complex lifestyle and psychological intervention in knee osteoarthritis: scoping review of randomized controlled trials. Int J Environ Res Public Health.

[29] Hausmann LRM, Youk A, Kwoh CK, Ibrahim SA, Hannon MJ, Weiner DK (2017). Testing a positive psychological intervention for osteoarthritis. Pain Med.

[30] Li Q, Amano K, Link TM, Ma CB (2016). Advanced imaging in osteoarthritis. Sports Health.

[31] Alghadir AH, Anwer S, Iqbal A, Iqbal ZA (2018). Test–retest reliability, validity, and minimum detectable change of visual analog, numerical rating, and verbal rating scales for measurement of osteoarthritic knee pain. J Pain Res.

[32] Vlaeyen JW, Kole-Snijders AM, Boeren RG, Van Eek H (1995). Fear of movement/(re) injury in chronic low back pain and its relation to behavioral performance. Pain..

[33] Crombez G, Eccleston C, Van Damme S, Vlaeyen JW, Karoly P (2012). Fear-avoidance model of chronic pain: the next generation. Clin J Pain.

[34] Herman T, Giladi N, Hausdorff JM (2011). Properties of the 'timed up and go' test: more than meets the eye. Gerontology..

[35] Dobson F, Hinman RS, Roos EM, Abbott JH, Stratford P, Davis AM, Buchbinder R, Snyder-Mackler L, Henrotin Y, Thumboo J, Hansen P (2013). OARSI recommended performance-based tests to assess physical function in people diagnosed with hip or knee osteoarthritis. Osteoarthr Cartil.

[36] Ries JD, Echternach JL, Nof L, Gagnon BM (2009). Test-retest reliability and minimal detectable change scores for the timed "up & go" test, the six-minute walk test, and gait speed in people with Alzheimer disease. Phys Ther.

[37] Zasadzka E, Borowicz AM, Roszak M, Pawlaczyk M (2015). Assessment of the risk of falling with the use of timed up and go test in the elderly with lower extremity osteoarthritis. Clin Interv Aging.

[38] Polit DF, Beck CT (2013). Essentials of nursing research: appraising evidence for nursing practice.

[39] Preece SJ, Brookes N, Williams AE, Jones RK, Starbuck C, Jones A, Walsh NE (2021). A new integrated behavioural intervention for knee osteoarthritis: development and pilot study. BMC Musculoskelet Disord.

[40] Lundberg M, Styf J (2009). Kinesiophobia among physiological overusers with musculoskeletal pain. Eur J Pain.

[41] Zampogna B, Papalia R, Papalia GF, Campi S, Vasta S, Vorini F, Fossati C, Torre G, Denaro V (2020). The role of physical activity as conservative treatment for hip and knee osteoarthritis in older people: a systematic review and meta-analysis. J Clin Med.

[42] Champagne A, Prince F, Bouffard V, Lafond D (2012). Balance, falls-related self-efficacy, and psychological factors amongst older women with chronic low Back pain: a preliminary case-control study. Rehabil Res Pract.

[43] Osajie FE, Yakubu K (2015). A retrospective non-comparative analysis of the quality of care for osteoarthritis at the general out-patient department of Jos University Teaching Hospital, Nigeria. J Fam Med Prim Care.

[44] Khalaj N, Abu Osman NA, Mokhtar AH, Mehdikhani M, Wan Abas WA (2014). Balance and risk of fall in individuals with bilateral mild and moderate knee osteoarthritis. PLoS One.

[45] Rantakokko M, Portegijs E, Viljanen A, Iwarsson S, Rantanen T (2017). Task modifications in walking postpone decline in life-space mobility among community-dwelling older people: a 2-year follow-up study. J Gerontol A Biol Sci Med Sci.

[46] Akinpelu AO, Alonge TO, Adekanla BA, Odole AC (2009). Prevalence and pattern of symptomatic knee osteoarthritis in Nigeria: a community-based study. Internet J Allied Health Sci Pract.

[47] Hootman JM, Helmick CG, Barbour KE, Theis KA, Boring MA (2016). Updated projected prevalence of self-reported doctor-diagnosed arthritis and arthritis-attributable activity limitation among US adults, 2015-2040. Arthritis Rheumatol.

[48] Elboim-Gabyzon M, Rozen N, Laufer Y (2012). Gender differences in pain perception and functional ability in subjects with knee osteoarthritis. ISRN Orthop.

[49] Prieto-Alhambra D, Judge A, Javaid MK, Cooper C, Diez-Perez A, Arden NK (2014). Incidence and risk factors for clinically diagnosed knee, hip and hand osteoarthritis: influences of age, gender and osteoarthritis affecting other joints. Ann Rheum Dis.

[50] Skantz H, Rantanen T, Palmberg L, Rantalainen T, Aartolahti E, Portegijs E (2020). Outdoor mobility and use of adaptive or maladaptive walking modifications among older people. J Gerontol A Biol Sci Med Sci.

